# Supplementary material for: ABCF protein-mediated resistance shapes bacterial responses to antibiotics based on their type and concentration
Source: mBio. 2025 Aug 12;16(9):e01568-25. doi: 10.1128/mbio.01568-25 (PMC12421871; doi:10.1128/mbio.01568-25)
Supplement: Data S5 — Full-size gels of ribosome toeprinting. [file mbio.01568-25-s0005.pdf]

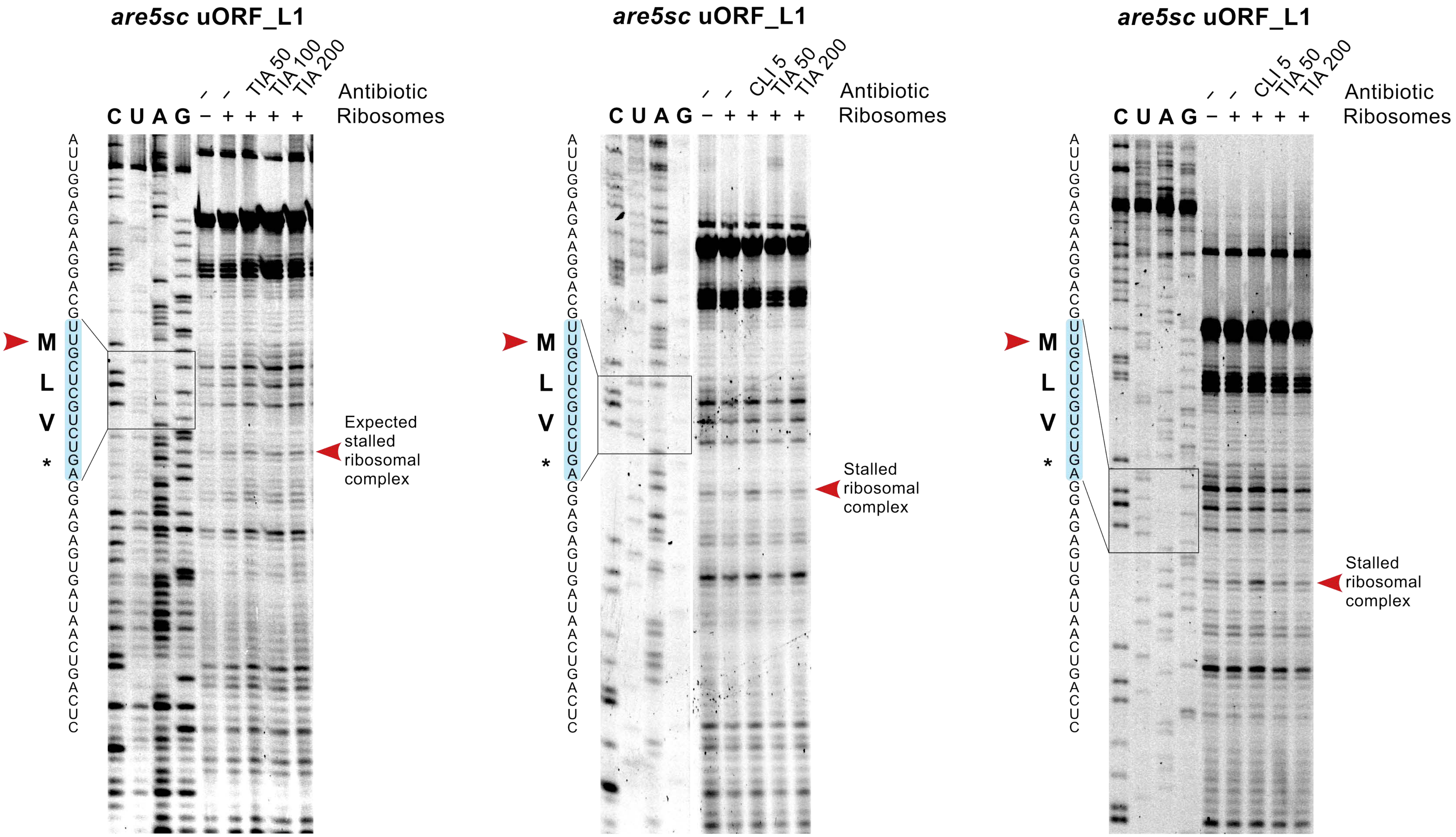

are5sc uORF_L1
TIA 50
TIA 100
TIA 200
Antibiotic
Ribosomes
C U A G − + + + +
AUUGGAGAAGGACGUUGCUCGUCUGAGGAGAGUGAUAACUGACUC
M
L
V
*
Expected stalled ribosomal complex
are5sc uORF_L1
CLI 5
TIA 50
TIA 200
Antibiotic
Ribosomes
C U A G − + + + +
Stalled ribosomal complex
are5sc uORF_L1
CLI 5
TIA 50
TIA 200
Antibiotic
Ribosomes
C U A G − + + + +
Stalled ribosomal complex

***are5sc* uORF_L1 (AUG$_{mut}$)**

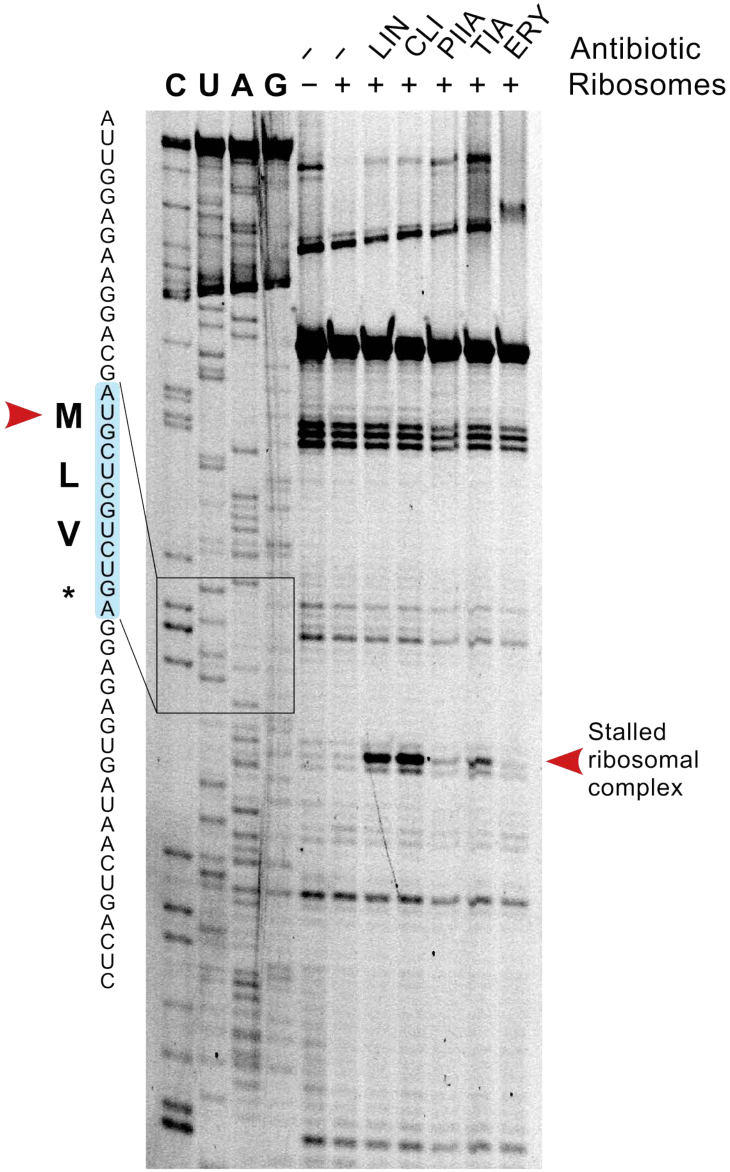

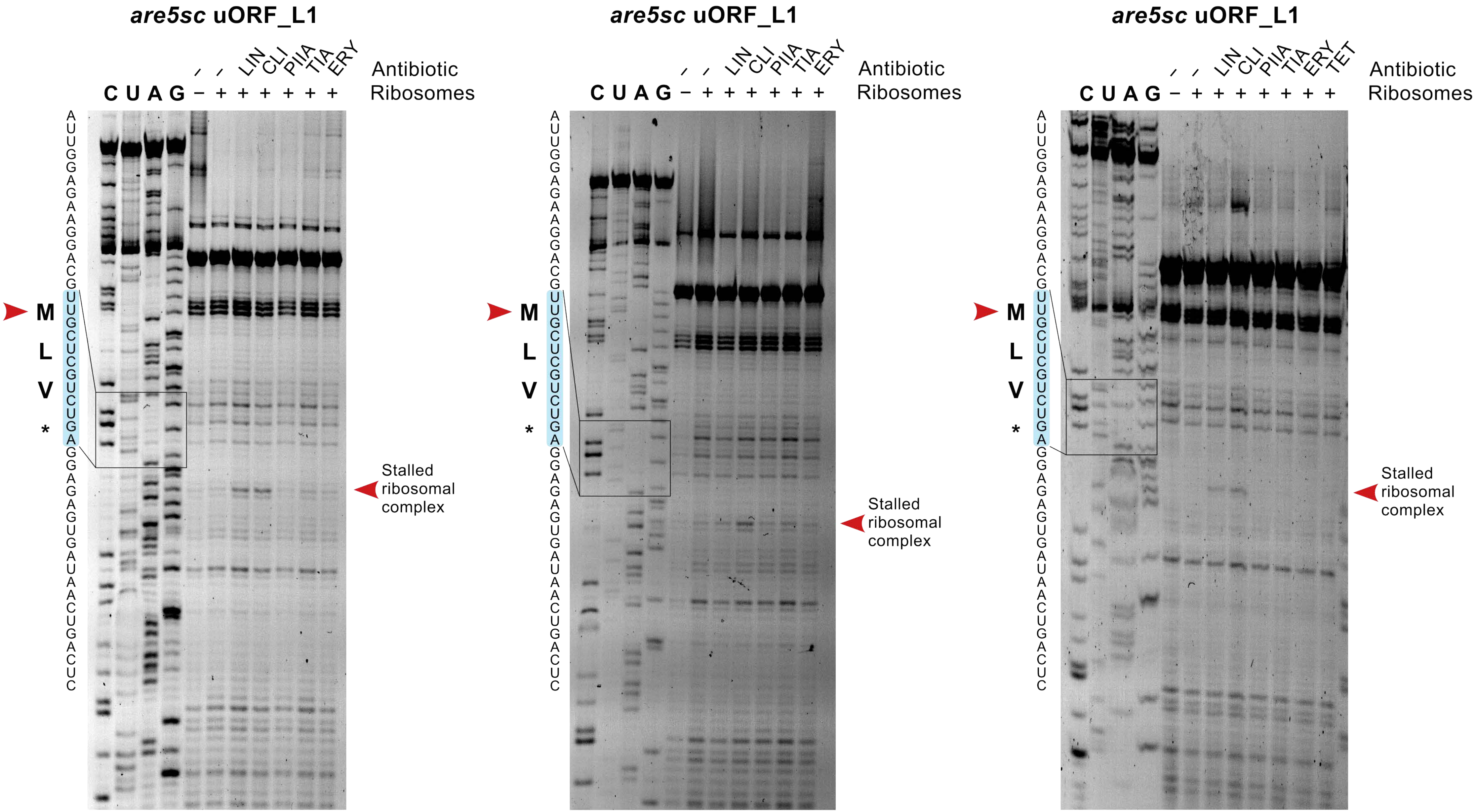
are5sc uORF_L1
– – LIN CLI PIIA TIA ERY
Antibiotic
C U A G – + + + + + +
Ribosomes
AUUGGAGAAGGACGUUGCUCGUCUGAGGAGAGUGAUAACUGACUC
M
L
V
*
Stalled ribosomal complex
are5sc uORF_L1
– – LIN CLI PIIA TIA ERY
Antibiotic
C U A G – + + + + + +
Ribosomes
AUUGGAGAAGGACGUUGCUCGUCUGAGGAGAGUGAUAACUGACUC
M
L
V
*
Stalled ribosomal complex
are5sc uORF_L1
– – LIN CLI PIIA TIA ERY TET
Antibiotic
C U A G – + + + + + + +
Ribosomes
AUUGGAGAAGGACGUUGCUCGUCUGAGGAGAGUGAUAACUGACUC
M
L
V
*
Stalled ribosomal complex

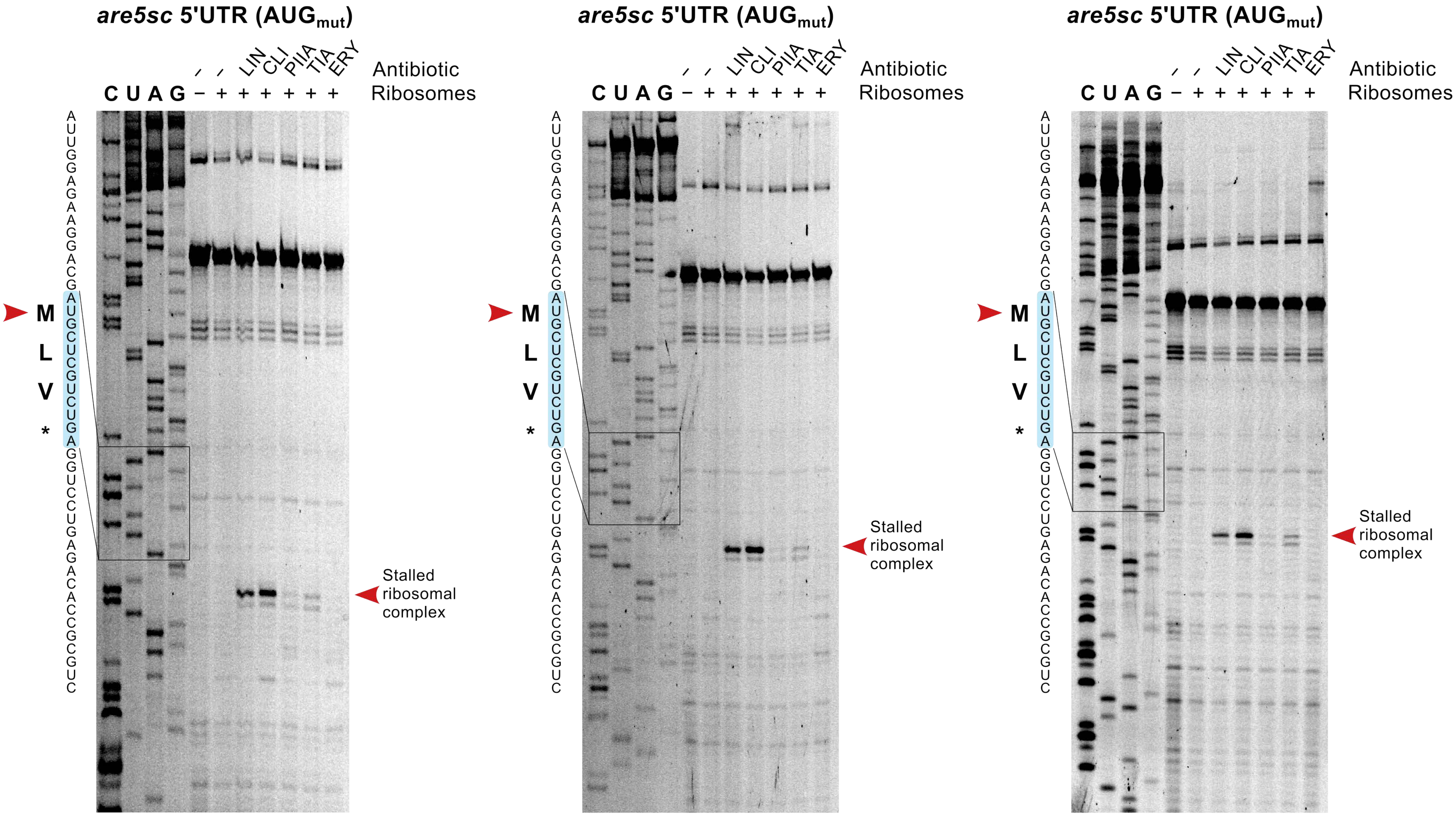

are5sc 5'UTR (AUG$_{mut}$)
C U A G
– + + + + + +
/ / LIN CLI PIIA TIA ERY
Antibiotic
Ribosomes
A U U G G A G A A G G A C G A U G C U C G U C U G A G G U C C U G A G A C A C C G C G U C
M
L
V
*
Stalled ribosomal complex
are5sc 5'UTR (AUG$_{mut}$)
C U A G
– + + + + + +
/ / LIN CLI PIIA TIA ERY
Antibiotic
Ribosomes
A U U G G A G A A G G A C G A U G C U C G U C U G A G G U C C U G A G A C A C C G C G U C
M
L
V
*
Stalled ribosomal complex
are5sc 5'UTR (AUG$_{mut}$)
C U A G
– + + + + + +
/ / LIN CLI PIIA TIA ERY
Antibiotic
Ribosomes
A U U G G A G A A G G A C G A U G C U C G U C U G A G G U C C U G A G A C A C C G C G U C
M
L
V
*
Stalled ribosomal complex

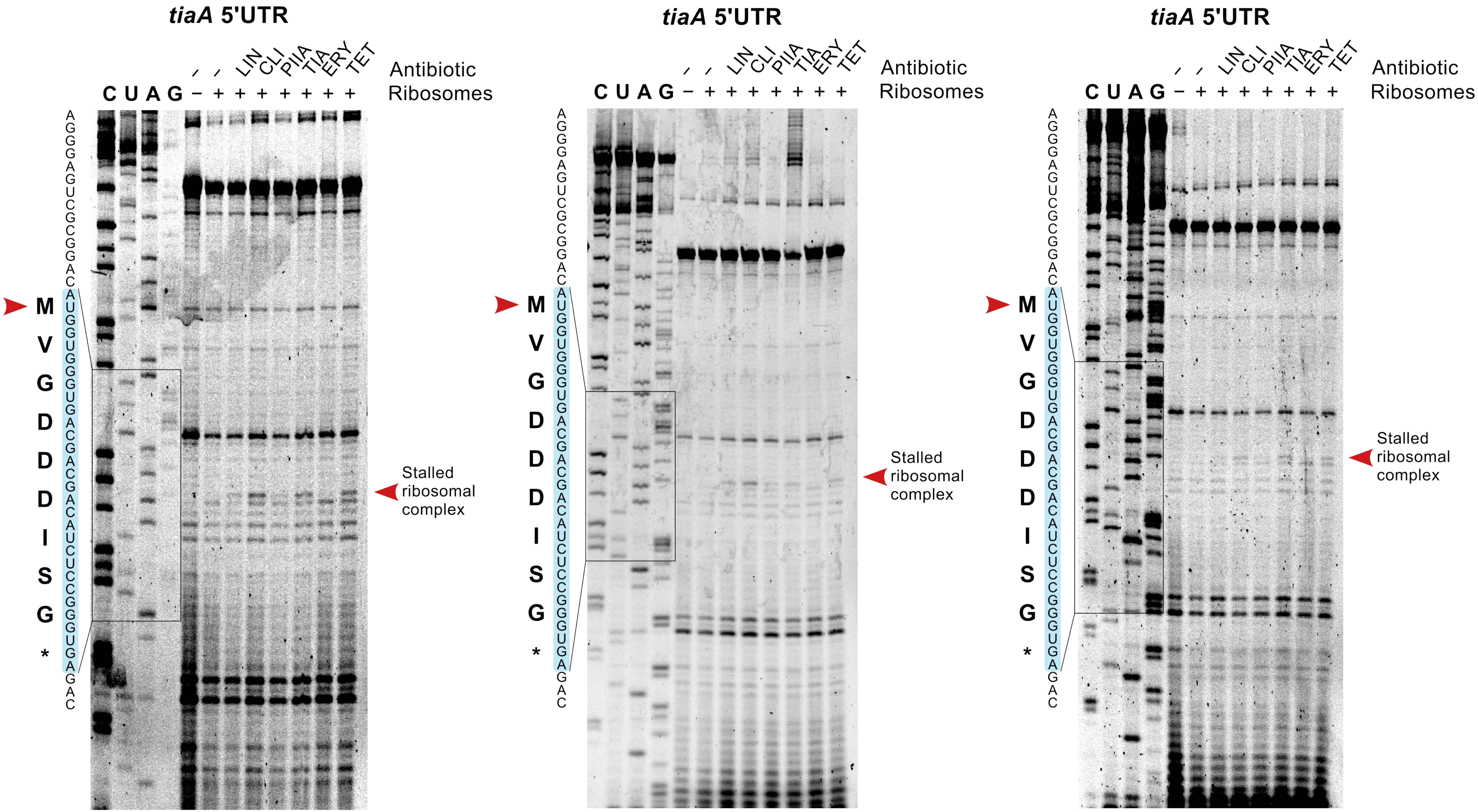

tiaA 5'UTR
LIN CLI PIIA TIA ERY TET
Antibiotic
C U A G − + + + + + + +
Ribosomes
AGGGAGUCGCGGAC
M V G D D D I S G *
AUGGUGGGUGACGACGACAUCUCCGGGUGA
GAC
Stalled ribosomal complex
tiaA 5'UTR
LIN CLI PIIA TIA ERY TET
Antibiotic
C U A G − + + + + + + +
Ribosomes
Stalled ribosomal complex
tiaA 5'UTR
LIN CLI PIIA TIA ERY TET
Antibiotic
C U A G − + + + + + + +
Ribosomes
Stalled ribosomal complex
